# Application of a Developed Method for the Extraction of Triazines in Surface Waters and Storage Prior to Analysis to Seawaters of Galicia (Northwest Spain)

**DOI:** 10.1155/2013/536369

**Published:** 2013-10-08

**Authors:** Noelia Rodríguez-González, Elisa Beceiro-González, María José González-Castro, Soledad Muniategui-Lorenzo

**Affiliations:** Department of Analytical Chemistry, Faculty of Science, A Coruña University, Campus da Zapateira, Rua da Fraga 10, 15008 A Coruña, Spain

## Abstract

A simple method based on solid-phase extraction combined with liquid chromatography for simultaneous determination of nine triazine herbicides (ametryn, atrazine, cyanazine, prometryn, propazine, simazine, simetryn, terbuthylazine, and terbutryn) in surface water samples was developed and validated. Under optimized conditions, 50 mL of water sample was pumped through the Oasis HLB cartridge, and triazines were eluted with 3 mL acetone. Finally the extract was concentrated to dryness, reconstituted with 1 mL methanol : water (1 : 1) and injected into the HPLC-DAD system. The stability of the herbicides on the cartridges at −18 and 4°C was also evaluated, and the recoveries obtained after three weeks of storage were satisfactory for all compounds. The analytical features of the proposed method were satisfactory: repeatability and intermediate precision were <10% and recoveries in spiked river water and seawater samples were higher than 93% for all compounds studied. Limits of quantification (varied from 0.46 to 0.98 *µ*g L^−1^) were adequately allowing the determination of these compounds at the levels requested by the 2008/105/EC Directive. Finally, this method was applied to the analysis of 50 seawater samples from Galicia (northwest Spain).

## 1. Introduction

Triazines are recognised herbicides which have been broadly used in agriculture over the recent decades. The surface water receives fluxes of these compounds mainly of agricultural origin, due to their widespread use in this field [1]. Their high persistence and toxicity have required rigorous control of environmental contamination. Therefore, the presence of pesticides in surface waters is regulated by the European Directive 2008/105/EC that establishes a maximum permitted concentration of 2 and 4 *μ*g L^−1^ for atrazine and simazine, respectively [2]. It is important to take into account that atrazine and simazine have been included in the list of “priority hazardous substances” in Decision 2455/2001/EC [3] that amends the Directive 2000/60/EC [4], and atrazine, ametryn, prometryn, terbutryn, simazine, and propazine are considered as a group to be endocrine-disrupting chemicals by the US Environmental Protection Agency [5]. Thus, sensitive methods for determining the low concentrations of triazine herbicides in environmental samples are required. 

Different chromatographic techniques have been used to determine triazines. Gas chromatography coupled to mass spectrometry has been widely employed [6–11], and the use of liquid chromatography with different detectors such as ultraviolet [12–16], diode array [17, 18], or mass spectrometry [19–25] has also been reported.

An extraction procedure to preconcentrate the analytes and remove possible interferences is mandatory to achieve the required levels. For this purpose, solid-phase extraction (SPE) is the preconcentration technique most commonly used for the determination of triazines in water samples [7–11, 17, 19–23, 25]. Recently, microextraction techniques have become important procedures in environmental analysis. Thus, some microextraction methods such as solid-phase microextraction (SPME) [26], stir bar sorptive extraction (SBSE) [6], liquid phase microextraction (LPME) [24], liquid-liquid-solid microextraction (LLSME) [13], and dispersive liquid-liquid microextraction (DLLME) [14, 18, 27] have been applied for extraction and preconcentration of triazines in water as alternative to the SPE techniques. However, some of these new techniques have drawbacks like low sensitivity for the triazines studied [26], poor recoveries [14, 18], and in many cases they are very laborious [13, 24].

The aim of the current study was to develop a simple, sensitive, and low-cost method based on SPE for the extraction of nine triazines from surface water samples and the storage of the compounds until their determination by HPLC-DAD. Samples of river water and seawater were chosen to illustrate the reliability of the method. Finally, the method was applied to analyze 50 samples of seawater from two areas dedicated to shellfishing and fishing. 

## 2. Experimental

### 2.1. Site Location and Sampling

**Figure 1:**
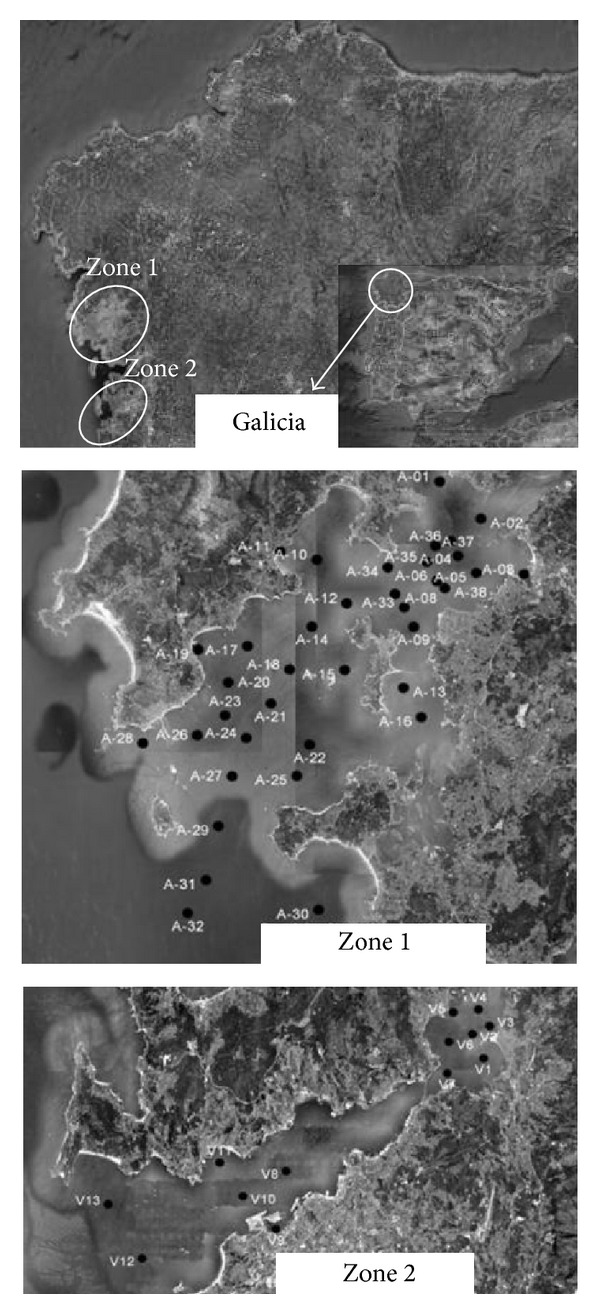
Map of sampling sites location at Galicia (NW Spain).

Fifty seawater samples from Galicia (northwest Spain) were collected at two different locations (zones 1 and 2) from April to June 2011. The health of both estuaries is a priority for the government of the region, because they are engaged in shellfish and fishing. In zone 1, (estuary of Arousa Island) 37 sites were selected for sampling and in zone 2, (estuary of Vigo) 13 sites were selected. The sampling locations and their designations are shown in Figure 1. At each sampling location three samples were collected.

Samples were collected in amber glass bottles and transported to the laboratory under cooled conditions (4°C). Upon reception, samples were filtered through 0.6 *μ*m glass fibre filters to eliminate suspended solid matter and the solid-phase extraction was carried out. The cartridges were stored at −18°C in the dark until analysis. 

### 2.2. Chemical and Materials

All herbicides analytical standards were supplied by Sigma-Aldrich (Inc. St. Louis, MO, USA). The individual stock standard solutions of 1000 mg L^−1^ were prepared in methanol by exact weighing of high-purity substances and stored at–18°C in the dark. Then a mixture of all the compounds was prepared in methanol containing 10 mg L^−1^ each individual triazine and stored at −18°C. All working solutions were daily prepared by appropriate dilution of the 10 mg L^−1^ standard solutions with methanol:water (1 : 1, v/v). 

Acetonitrile was purchased from Panreac (Barcelona, Spain) and methanol and acetone from Romil (Cambridge, UK). All chemicals were of HPLC grade. Ultra-pure Milli-Q water was obtained using a Millipore Milli-Q system (Millipore, Bedford, MA, USA).

Reverse phase polymeric cartridges Oasis HLB (6 mL, 200 mg) were supplied by Waters (Milford, MA, USA). Glass fibre MN GF-6 filters (0.6 *μ*m pore size) were purchased from Macherey Nagel (Düren, Germany) and 0.20 *μ*m pore-size nylon membrane filters from Millipore (Bedford, MA, USA).

A visiprep vacuum system from Supelco (Bellefonte, PA, USA), a rotary evaporator (Büchi, Labortechnic AG, Flawil, Switzerland), and an ultrasonic bath (Branson 3200, Energieweg, The Netherlands) were used.

Uncontaminated river and seawater samples collected from a brook and Riazor beach in the city of A Coruña (Galicia, NW Spain) were used for the validation of the method. 

### 2.3. Instrumental Analysis

Chromatographic analyses were performed using a high performance liquid chromatography-diode array detector (HPLC-DAD). The system consisted of a 2695 pump with a 996 Diode Array Detector from Waters (Milford, MA, USA). The column was a stainless steel column (150 mm × 4.6 mm ID, particle size 5 *μ*m) packed with Hypersil GOLD C_18_ chemical bonded phase from Thermo Scientific (Austin, TX, USA). 

The analysis was carried out using the following gradient elution: acetonitrile initial percentage of 30% (8 min) increased linearly to 40% in 5 min and increased to 50% in 5 min, after which the percentage was returned to the initial conditions in 9 min. The flow rate was 1 mL min^−1^, the oven temperature was set at 25°C, and 20 *μ*L of sample volume was used.

The absorbance was measured continuously in the 200–400 nm range, and peaks areas quantification were carried out at 222.7 nm in order to achieve maximum sensitivity.

All triazine herbicides were identified initially by retention time and then by applying spectral contrast techniques (incorporated in Millenium^32^ software) homogeneity of the spectral peak was confirmed. Finally, a spectral identification was carried out contrasting the spectrum with a standard library created in wavelength interval of 200–400 nm.

### 2.4. Solid-Phase Extraction Procedure

The extraction was performed as follows: the cartridge was conditioned by washing it with 10 mL methanol and 10 mL Milli-Q water. Water sample (50 mL) was pumped through the cartridge at a flow rate of 10 mL min^−1^ and then the cartridge was washed with 20 mL Milli-Q water. Once the retention step had been completed, the cartridge was partially dried under a vacuum system for 5 min and then it was totally dried using a nitrogen stream for 30 min. The elution of retained compounds was done with 3 mL of acetone, and the organic extract was brought to complete dryness under a combination of rotary evaporator at 40°C and a gentle nitrogen stream. Finally the sample was reconstituted in methanol/water (1 : 1, v/v) to a final volume of 1 mL and injected into the HPLC. 

## 3. Results and Discussion

### 3.1. SPE Method Optimization

The solid-phase extraction procedure was based on a previous method for drinking water developed by the same authors [28]. To optimize the method for surface waters, a filtration step prior to the SPE was studied. For this purpose, two different filters were assayed (glass microfiber filters and nylon membrane filters). A volume of 50 mL of seawater sample was spiked at a concentration level of 2 *μ*g L^−1^ (the lowest legislation level). The spiked sample was mixed in an ultrasonic bath for 5 min to ensure efficient distribution of the herbicides, and it was allowed to equilibrate for 5 min prior to extraction, and then it was filtered. Isolation and determination of the compounds from the spiked samples were performed as described above.

Results showed an important decrease on the recovery for simetryn (68%) and terbutryn (50%) when nylon membrane filter was employed (recoveries between 82 and 101% were obtained for the other seven herbicides). However, values of recovery were satisfactory for all compounds when glass microfiber filter was used. 

### 3.2. Stability of Herbicides on the Cartridges

Due to that herbicides may be degraded during storage of the sample at 4°C by processes such as hydrolysis or microbial decomposition, the fact of being able to perform the storage of the cartridges after the solid-phase extraction for extend time periods is extremely useful. Thus, the extraction can be performed on the day of sampling or even at the time of sampling for further transport to the laboratory. For this reason, the stability of the herbicides loaded on Oasis HLB cartridges was investigated under different storage conditions. Several cartridges were loaded with 50 mL of seawater spiked at 2 *μ*g L^−1^ with a standard mixture of the nine herbicides and stored at 4 and −18°C. Elution of triazines was carried out after 1, 2, 3, 6, and 8 weeks of storage employing three cartridges for each time and temperature studied. Before elution, cartridges kept at –18°C and 4°C were defrosted at room temperature for 2 and 1 h, respectively. The organic extracts obtained were reconstituted and analyzed. 

**Figure 2:**
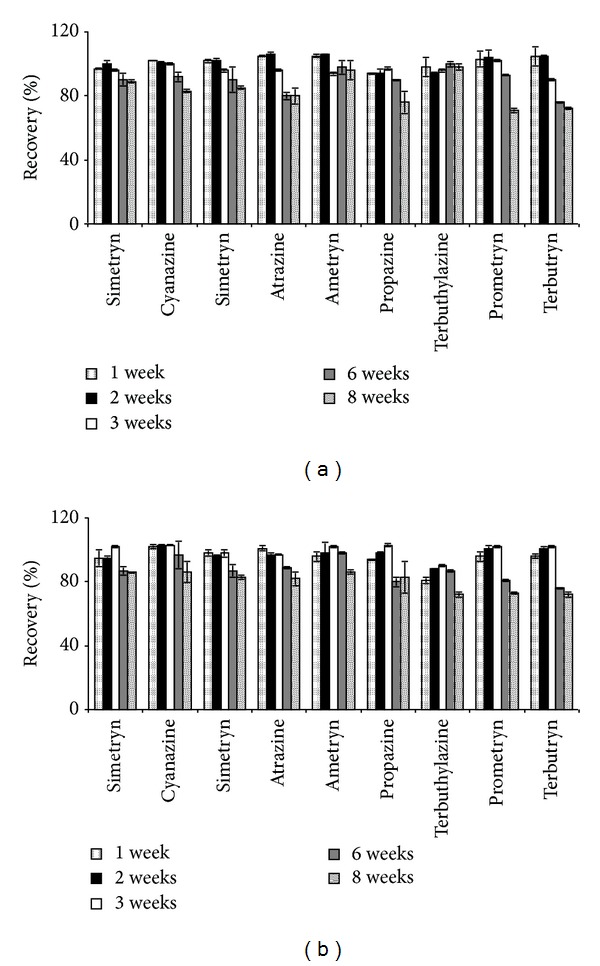
Effect of storage conditions on the recovery of the herbicides. (a) Temperature −18°C, (b) temperature 4°C.

The effect on the recovery of the herbicides was evaluated. The results are shown in Figure 2. As can be seen (Figure 2(a)), triazines showed a high stability at −18°C being the recoveries quantitative after 6 weeks of storage for all the compounds (recovery values higher than 90% and RSD < 10%), except atrazine and terbutryn in which case recovery considerably decreased after 3 weeks (80 and 76%, resp., after 6 weeks of storage). However, the recovery of the compounds was only quantitative up to 3 weeks at 4°C, and it was decreasing gradually for higher storage periods of time, mainly for prometryn, propazine, and terbutryn (Figure 2(b)). On the other hand, a major variability on the recoveries was observed at 4°C. Therefore, it can be concluded that the most reliable method for storing the herbicides on the cartridge is keep them at –18°C during 3 weeks because the integrity of the analytes is not affected. 

### 3.3. Method Validation

The analytical characteristics of the SPE-HPLC method were evaluated using a 50 mL of uncontaminated seawater sample spiked with a standard mixture of the compounds. The linearity was studied at 6 concentration levels (0.5, 1, 1.5, 2, 2.5, and 3 *μ*g L^−1^). As can be seen in Table 1, determination coefficients (*r* ^2^) were higher than 0.991 for all the herbicides at concentrations within the interval tested. 

The limits of detection (LODs) were determined as 3∗*S*_*y*/*x*_/*b* and the limits of quantification (LOQs) as 10∗*S*_*y*/*x*_/*b*, where *S*_*y*/*x*_ is the residual standard deviation and *b* is the slope of the calibration curves. As can be seen in Table 1, the detection and quantification limits between 0.15–0.33 and 0.46–0.98 *μ*g L^−1^, respectively, were adequate, being the LOQs much lower than parametric value requested by the legislation for surface water [2].

Repeatability and intermediate precision were evaluated at 2 *μ*g L^−1^. The repeatability was calculated as within-day RSD of peak areas using eight replicates analyzed in the same day and by the same analyst. In the case of intermediate precision, five replicates were analyzed in consecutive days and by the same analyst and it was calculated as between-day RSD of peak areas. As can be seen in Table 1, the results obtained were satisfactory with RSD values below 10% for all compounds in both cases. 

The accuracy (expressed as percent recovery) of the method was studied using seawater sample spiked with 2 *μ*g L^−1^ of a standard mixture of the compounds. The recoveries obtained for five replicates (*n* = 5) of the sample spiked with the triazine herbicides are presented in Table 2. The results demonstrated that the method achieved satisfactory recoveries in the range of 93%–106%, with associate standard deviations below 9% for all compounds. 

Furthermore, a river water sample was also used to evaluate the reliability of the SPE-HPLC method. For this purpose, five replicates of 50 mL of sample spiked at 2 *μ*g L^−1^ with a standard mixture of the compounds were subjected to the optimized method and analytical recoveries were evaluated. The results obtained (see Table 2) have been shown to be satisfactory with recoveries in the range of 95%–104%, with RSD below 7% for all compounds. 

**Figure 3:**
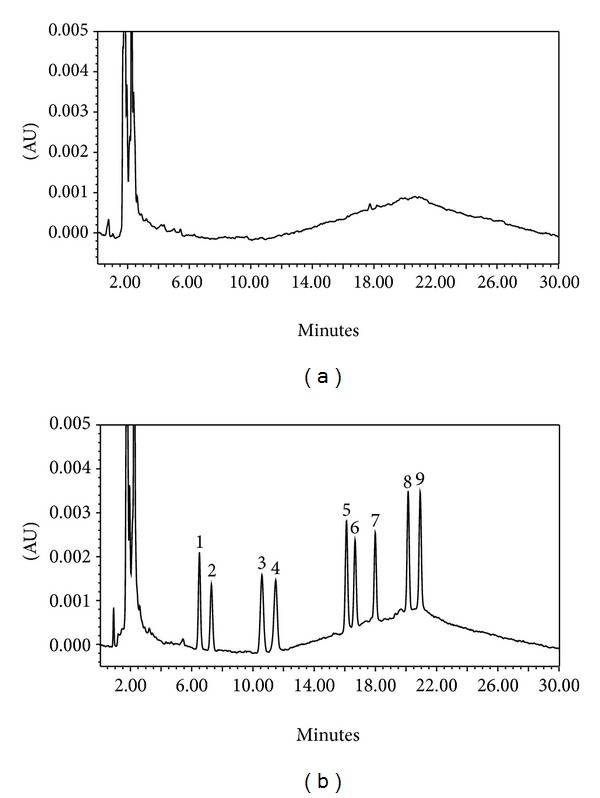
Chromatograms obtained after solid-phase extraction. (a) seawater sample, (b) seawater sample spiked at 2 *μ*g L^−1^. Target compounds are numbered as follows: (1) simazine, (2) cyanazine, (3) simetryn, (4) atrazine, (5) ametryn, (6) propazine, (7) terbuthylazine, (8) prometryn, and (9) terbutryn.

Therefore, it can be concluded that the proposed method is useful to determine triazines in surface water samples. As an example, chromatograms corresponding to seawater (Figure 3(a)) and seawater sample spiked at a concentration level of 2 *μ*g L^−1^ (Figure 3(b)) are presented in Figure 3. 

### 3.4. Application

Finally, the method was applied to the analysis of 50 seawater samples from Galicia (NW Spain). Although the herbicides under study have not been detected in the samples analyzed, studies to generate information related to their levels in areas where seafood is growing (mussels, crabs, oysters,…) are of great economic and environmental interest. 

## 4. Conclusion

The proposed method provides a simple and inexpensive way for simultaneous determination of nine triazine herbicides in surface waters. Furthermore, it uses small volume of organic solvents in agreement with the principles of Green Chemistry. The stability of the herbicides on the cartridge stored at −18°C is of great interest and usefulness since the use of the cartridges allows the storage of the triazines until analysis, avoiding the problem associated with the maintaining herbicides integrity in aqueous solution when long periods of storage are required before analysis. This fact makes the solid-phase extraction procedure developed as a promising alternative to conventional water sampling for triazines analysis. However, further work should be necessary to optimize the extraction methodology “in situ” during sampling. 

The method was successfully applied for river and seawater samples, and satisfactory precision, accuracy, and sensitivity were obtained. Using 50 mL of seawater sample, the LOQs obtained were lower than the parametric value requested by the legislation [2].

The method was applied to the analysis of 50 seawater samples from Galicia (NW Spain). Although the triazines under study have not been detected in the samples analyzed, the monitoring of their levels in marine ecosystems is of great economic and environmental importance. It is important to take into account that measurements of known quality represent the foundation of the water quality evaluation system and the basis for decisions to be taken to achieve the Marine Strategy Framework Directive and environmental objectives at the end of 2015 [29]. 

Finally, it is noteworthy that methods based on solid-phase extraction combined with liquid chromatography have been commonly used to measure triazines in drinking and ground waters; however, there are not studies in seawater.

## Figures and Tables

**Table 1:** Analytical characteristics of the SPE-HPLC method.

Compound	LOD (*μ*g L^−1^)	LOQ (*μ*g L^−1^)	Determination coefficient (*r* ^2^)	Repeatability^a^ RSD (%)	Reproducibility^a^ RSD (%)
Simazine	0.28	0.84	0.9962	4.3	10.0
Cyanazine	0.33	0.98	0.9910	4.4	4.8
Simetryn	0.23	0.70	0.9955	5.7	6.5
Atrazine	0.15	0.46	0.9981	5.5	7.6
Ametryn	0.28	0.85	0.9915	5.2	5.2
Propazine	0.18	0.56	0.9978	4.5	8.6
Terbuthylazine	0.26	0.80	0.9948	7.6	8.3
Prometryn	0.16	0.50	0.9982	1.4	2.4
Terbutryn	0.26	0.80	0.9966	3.1	5.1

^a^ *n* = 8 and *n* = 5 for repeatability and reproducibility respectively (2 *μ*g L^−1^).

**Table 2:** Study of analytical recovery in river and sea waters (2 *μ*g L^−1^, *n* = 5).

Compound	Analytical recovery (%) ± RSD (%)	
	River water	Seawater
Simazine	100 ± 7.0	100 ± 7.8
Cyanazine	100 ± 6.1	106 ± 8.8
Simetryn	100 ± 3.1	103 ± 9.0
Atrazine	96 ± 5.3	93 ± 8.9
Ametryn	100 ± 5.1	102 ± 7.3
Propazine	99 ± 5.4	103 ± 8.7
Terbuthylazine	104 ± 2.5	99 ± 8.4
Prometryn	96 ± 6.6	106 ± 6.4
Terbutryn	95 ± 4.1	99 ± 2.3
